# Congestive heart failure and concealed accessory pathway conduction in a patient with complete heart block

**DOI:** 10.1016/j.hrcr.2022.05.002

**Published:** 2022-05-13

**Authors:** Erden Goljo, Roger Fan

**Affiliations:** Division of Cardiology, Department of Medicine, Stony Brook University, Stony Brook, New York

**Keywords:** Electrophysiology, Ablation, Heart failure, Accessory pathway, Wolff-Parkinson-White

**Key Teaching Points**

• Very late accessory pathway conduction can recur after initially successful ablation.
• Heart failure can be a consequence of tachycardia or dyssynchrony from manifest preexcitation, but rarely—as this case demonstrates—may also be from atrial ectopy from concealed accessory pathway conduction.
• Definitive ablation is the recommended treatment for patients with congestive heart failure related to accessory pathway conduction.

## Introduction

Late recurrence of successful accessory pathway (AP) ablation in adults is rare. We report a case of a patient with prior ablation for Wolff-Parkinson-White (WPW) syndrome who presented with acute decompensated heart failure in the setting of pacing-related ectopy over a concealed recurrent AP.

## Case report

A 40-year-old male patient with WPW syndrome with a right anteroseptal AP who underwent multiple ablations (ultimately successful 7 years prior), which were complicated by complete heart block requiring single-chamber VDD pacemaker, presented to our hospital with 2 weeks of progressive dyspnea on exertion and lower-extremity edema and was admitted for acutely decompensated congestive heart failure.

**Figure 1:**
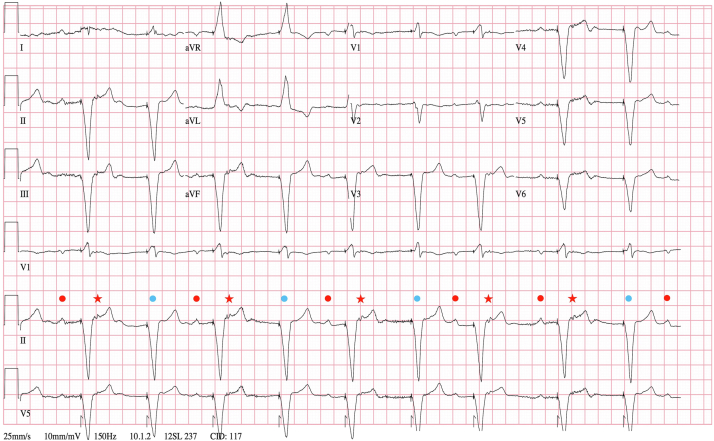
Admission electrocardiogram showing normal sinus rhythm (*red dots*) with ventricular tracking and bigeminal atrial premature beats (*stars*), followed by sinus pause with demand ventricular pacing likely coinciding with the next sinus p wave (*blue dots*).

**Figure 2:**
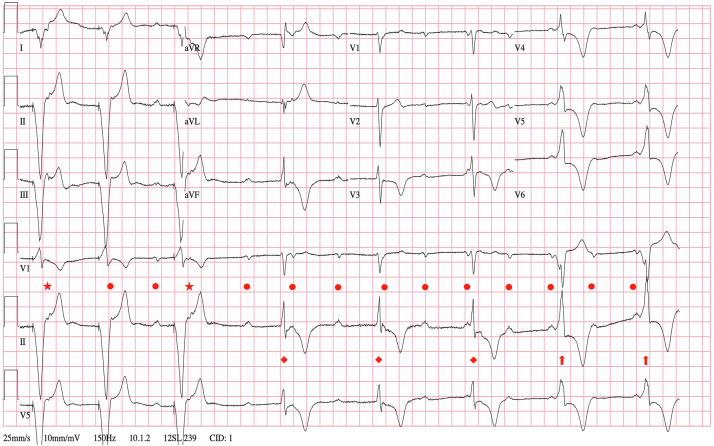
Electrocardiogram initially showing paced rhythm from Figure 1 progressing to complete heart block with junctional escape rhythm owing to VDD pacemaker oversensing, which then transitions to 2:1 A-V conduction via accessory pathway. Dots = sinus rhythm; Stars = premature atrial beats; Diamonds = junctional escape rhythm; Arrows = accessory pathway A-V conduction.

Electrocardiogram on admission showed normal sinus rhythm with ventricular tracking and atrial premature beats in a bigeminal and trigeminal pattern (Figure 1). Unexpectedly he had occasional periods of normal sinus rhythm with 1:1 ventricular preexcitation. Pacemaker interrogation on admission revealed underlying complete heart block and was notable for 98.2% ventricular pacing and 5.6% mode switch burden over the preceding 7 months, compared to 96.7% ventricular pacing and 0.8% mode switch burden in the 4 years before that. Echocardiogram revealed his ejection fraction (EF) to be newly reduced to 40% from 55% 7 months prior. Regadenoson nuclear stress testing was normal without any evidence of ischemia. He was successfully diuresed and started on goal-directed medical therapy. The patient continued to have intermittent preexcitation as well as periods of junctional bradycardia to 40 beats per minute (Figure 2), thought to be due to pacemaker ventricular oversensing. Repeat device interrogation showed 55.1% ventricular pacing and 4.5% mode switch burden during the 4 days since admission.

**Figure 3:**
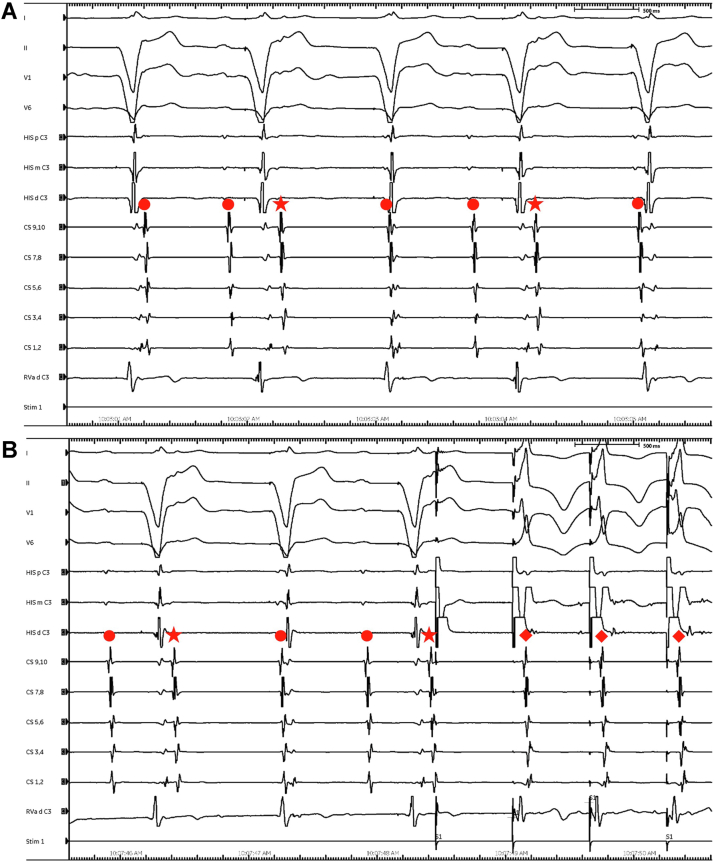
**A:** Normal sinus rhythm (*dots*) with complete heart block and VVI pacing at 60 beats/min, with frequent atrial ectopy with concentric coronary sinus activation and fixed VA timing (*stars*). **B:** Ventricular overdrive pacing showing robust 1:1 retrograde atrial activation (*diamonds*).

The patient underwent electrophysiological (EP) study (Figure 3). He presented in complete heart block and his pacemaker was set to VVI 60 beats per minute. Given frequent coughing and movement, he was placed under general anesthesia. Baseline electrogram showed ventricular pacing with what was initially believed to be frequent atrial premature beats with concentric coronary sinus activation and fixed VA timing (Figure 3A). However, ventricular overdrive pacing revealed robust retrograde 1:1 conduction down to 280 ms, with similar atrial activation sequence as the atrial ectopy (Figure 3B). Parahisian pacing and apical/base pacing both confirmed presence of retrograde AP conduction. Atrial and ventricular programmed electrical stimulation failed to induce supraventricular tachycardia. Adenosine failed to induce anterograde AP conduction. While right ventricle (RV) pacing, the earliest atrial activation was retrogradely mapped to the RV anteroseptum. Radiofrequency ablation immediately resulted in retrograde conduction block. No further atrial ectopy was seen. The pacemaker was reprogrammed to VDI 60.

The patient returned to the EP lab the following day for device revision owing to atrial undersensing and ventricular oversensing of the VDD lead, in addition to cardiac resynchronization therapy-pacemaker (CRT-P) upgrade. At that time, he was noted to be in normal sinus rhythm without any further atrial ectopy or mode switch episodes. Transthoracic echocardiography performed 8 months after CRT-P implantation showed EF improvement to 50%. Device interrogation 1 year after implantation revealed 100% biventricular pacing and no further mode switch episodes.

## Discussion

This patient with WPW syndrome over right anteroseptal AP, who underwent distant prior ablation complicated by complete heart block, presented with new-onset heart failure with new low EF of 40% and was found to have frequent atrial ectopy and intermittent return of preexcitation. We believe the etiology of his heart failure was multifactorial, but a significant component was owing to the high ectopic atrial burden from ventricular pacing–induced retrograde conduction over his concealed AP.

Cardiomyopathy due to ventricular dyssynchrony from ventricular preexcitation has previously been described.^1^, ^2^, ^3^ However, in this case, we feel the intermittent return of preexcitation likely plays a minor role in his congestive heart failure (CHF). He had a persistently preserved EF with manifest preexcitation for many years before his last ablation 7 years prior. Additionally, pacemaker interrogation on admission revealed 98.2% ventricular pacing over 7 months, but 55.1% during 4 days in the hospital, suggesting that the return of antegrade AP conduction was a recent phenomenon, following symptom onset and presentation. RV pacing dyssynchrony is also unlikely to be a primary etiology of CHF, since he has been pacer dependent for 7 years with documented preserved EF as recently as 7 months prior to presentation. However, it is possible that in the setting of the new atrial ectopic burden, both antegrade AP conduction and RV pacing synergistically impaired cardiac function.

The overall recurrence rate of AP conduction after ablation has been reported to be around 10% in multiple observational studies.^4^^,^^5^ Studies of long-term follow-up after AP ablation in adults are limited, but 1 study in a pediatric population^6^ found an overall recurrence rate after successful ablation to be similar to the general population (10.8%), with long-term recurrence after 1 year to be 2.2%.

In this case we believe that the patient had durable antegrade and retrograde AP block for 7 years after ablation but, at the time presentation, had selective return of retrograde AP conduction, manifested by ventricular pacing–induced retrograde atrial activation. Similarly, only close to the time of admission did antegrade AP conduction return, supported by the 98.2% pacing burden over the 7 months prior to admission but 55.1% pacing burden during 4 days of admission. The mechanism of intermittent anterograde AP conduction in this case is unknown. Conduction across a nondecremental AP is typically unmasked via the administration of adenosine, as the primary mechanisms of concealment are competing AV node conduction and retrograde invasion of the AP. Furthermore, adenosine has been shown to unmask dormant AP conduction immediately postablation by enhancing AP excitability.^7^ The patient in this report was given adenosine during EP study, with no unmasking of AP conduction. Given that he has permanent AV block and did not receive ablation prior to adenosine challenge, the significance of this finding is unclear. It is theoretically possible that the refractory period of the antegrade AP was consistently reset by RV pacing and either concealed or manifest retrograde atrial activation. Infusion of isoproterenol to shorten the AP refractory period during adenosine administration may have provided evidence to support this; however, this maneuver was not performed.

The ventricular pacing–induced retrograde atrial activation occurred in a bigeminal and trigeminal pattern owing to the programmed VDD pacing. The pacing-induced ectopy resulted in a sinus node pause, with ventricular demand pacing coinciding with the following sinus beat, rendering the concealed AP refractory. The following sinus beat and triggered ventricular pacing with retrograde atrial activation then reset the cycle (Figure 1). The atrial ectopy likely led to the 5.6% mode switch burden, which probably underrepresented the true burden, as the sinus p waves often fell in the blanking period, especially since the patient was observed to be predominantly in atrial bigeminy and trigeminy on telemetry during his hospitalization despite a 4.5% mode switch burden during that time. It is unlikely that the patient had true atrial fibrillation, as no episodes had previously been recorded on device interrogation, and after successful concealed AP ablation, at 1 year follow-up, no further mode switches were recorded on the device. The patient had recovery of EF to 50% after ablation. We believe this is primarily owing to resolution of atrial ectopy.

The contribution of CRT to recovery cannot be excluded. The patient required device revision owing to VDD lead atrial undersensing and ventricular oversensing. To minimize potential need for reoperation, we decided to upgrade to CRT-P at the same time. In retrospect we could have initially kept him programmed RV pacing only to assess EF improvement with resolution of atrial ectopy from AP ablation only. However, at the time we decided to program him with CRT pacing from the outset to maximize his recovery. As previously mentioned, we believe it less likely that RV dyssynchrony was the primary etiology of his CHF, as he had tolerated RV pacing for the last 7 years with preserved EF, as well as dyssynchrony from his manifest anteroseptal preexcitation for the 33 years preceding ablation.

## Conclusion

We present a rare case of very late recurrence of AP conduction after ablation. In addition, to our knowledge, this is the first described case of cardiomyopathy and CHF associated with pacing-induced retrograde atrial conduction over a concealed AP.
